# Efficacy of Three Doses of Halquinol on Growth Performance, Diarrhea Incidence, Nutrient Digestibility, and Fecal Microbiome of Weaned Pigs

**DOI:** 10.3390/ani15091258

**Published:** 2025-04-29

**Authors:** Panumas Kongpanna, Uttra Jamikorn, Thitima Tripipat, Angkana Tantituvanont, Rakthai Ngampak, Dachrit Nilubol

**Affiliations:** 1 Department of Animal Husbandry, Faculty of Veterinary Science, Chulalongkorn University, Bangkok 10330, Thailand; panumaskongpanna@gmail.com (P.K.); uttra.j@chula.ac.th (U.J.); 2 Swine Viral Evolution and Vaccine Development Research Unit, Department of Veterinary Microbiology, Faculty of Veterinary Science, Chulalongkorn University, Bangkok 10330, Thailand; tripipatt@gmail.com; 3 Department of Pharmaceutics and Industrial Pharmacy, Faculty of Pharmaceutical Sciences, Chulalongkorn University, Bangkok 10330, Thailand; angkana.t@pharm.chula.ac.th; 4 Department of Livestock Development, Ministry of Agriculture and Cooperatives, Bangkok 10400, Thailand; rakthairakthai@yahoo.com

**Keywords:** growth performance, halquinol, microbiome, nutrient digestibility, weaned pig

## Abstract

The weaning period is a critical phase for nursery pigs that is characterized by rapid growth and alterations in the intestinal microbiome associated with nutrient utilization. The present study aimed to investigate the efficacy of halquinol, when used as an antibiotic (ABO), on the growth performance, diarrhea incidence, coefficient of apparent total tract digestibility (CATTD), fecal volatile fatty acids (VFAs), and microbiota in pigs. A total of 210 healthy weaned pigs with an average initial weight of 6.9 kg and aged 28 ± 2 days were assigned to five treatments (six pens/treatment) in a complete randomized design, including a control group (T1, CON; feed with no ABO), a colistin group (T2, CLT; feed containing 120 ppm colistin), and three halquinol groups (T3 to T5, HAL; feed containing 180, 240, and 360 ppm halquinol, respectively). The experiment period lasted for 10 days. Field recordings, observation, and feces collection were performed on D1, D5, and D10. CATTD and VFA assessments were conducted on D10. The composition of the fecal microbiota was analyzed via 16S rRNA gene sequencing using the Illumina Miseq platform. The results demonstrated that the in-feed ABO groups exhibited a significantly lower ADFI (*p* < 0.01). Pigs fed the T3 and T4 diets had the lowest FCR (*p* < 0.01) on D5 and D10 and, thus, had reduced ADFI (*p* < 0.01). A quadratic contrast was found in ADFI and FCR on D5 and D10, indicating a negative correlation with HAL concentration (*p* < 0.01). Pigs fed CLT and HAL had significantly reduced levels of coliform (*p* < 0.01) and *E. coli* (*p* < 0.01). Moreover, pigs receiving ABO also had a lower fecal score compared to those on the CON diet (*p* < 0.01). Dietary in-feed ABO had no effect on all the parameters of the CATTD on D10 (*p* > 0.05), except for fat digestibility in pigs that received T4 (*p* < 0.01). Pigs fed the T4 and T5 diets had higher propionate concentrations and lower A/P ratios than pigs fed T1, T2, and T3 (*p* < 0.01). The microbial diversity shifted quickly through the early weaning period. The relative abundance of beneficial *Enterococcus* microbes increased in pigs fed in-feed ABO, whereas the relative prevalence of pathogenic bacteria, such as *Escherichia* and *Klebsiella*, decreased. *Escherichia* and *Bacteroides* were negatively correlated with carbohydrate digestibility and butyric and valeric acid production (*p* < 0.05). Overall, the appropriate HAL dosage was 240 ppm (T4), and this antimicrobial can potentially be characterized as an in-feed colistin replacer that improves feed efficiency and fat digestion, enhancing VFA production, alleviating post-weaning diarrhea, and protecting ABO-resistant piglets.

## Simple Summary

This study of halquinol provides an informative dataset, including information on the nutrient digestibility, fecal volatile fatty acid fermentation, and microbiome structure in pigs. Halquinol can serve as an alternative antibiotic in nursery pigs by preserving digestive capacity, stimulating enzymatic digestion and improving subsequent nutrient utilization. Although no significant differences in growth performance were observed between experimental diets, a reduced bacterial diversity in the microbiomes of halquinol-treated pigs might have contributed to an improvement in energy utilization. Understanding the development of pig microbiota during weaning, and its modulation in response to colistin and halquinol, is essential to designing alternative formulations for rational drug use.

## 1. Introduction

Globally, post-weaning diarrhea (PWD) is an economically important challenge in pig production. The post-weaning period involves several stressful situations for pigs, including a sudden change in the composition and physical form of the diet, separation from the sow, new social groups, and new environment adaption. During the two weeks following weaning, PWD can affect pigs and reduce feed intake, causing poor growth, diarrhea, and mortality [1]. These factors contribute to the growth of pathogens in the gastrointestinal tract (GIT), primarily enterotoxigenic strains of *Escherichia coli* (*E. coli*), which are the main causative agents.

The use of colistin as an in-feed antibiotic (ABO) has been effective at alleviating weaning stresses and preventing PWD in piglets. However, although colistin use is prohibited in Europe, it is still used in several nations around the world, notably those in Asia. In 2017, Thailand restricted the long-term use of colistin for the prevention of Gram-negative bacterial infections in animal feed, but it is still permitted for short-term treatments. Consequently, in 2018, the Department of Livestock Development (DLD) restricted the use of colistin for disease prevention in livestock and replaced it with halquinol. Practically, the commercial diets for weaned pigs contain multiple in-feed ABOs, including 300, 300, and 120 ppm of chlortetracycline, amoxycillin, and halquinol, respectively, to treat and inhibit diseases. Nevertheless, the inappropriate and excessive use of ABOs contributes to the widespread emergence and spread of antimicrobial resistance in livestock farms.

Halquinol (trade name Quixalud^®^) is an antimicrobial used as an in-feed ABO in poultry and swine. In swine, it is used as a growth promotant and for the treatment of intestinal infections caused by *E. coli* and *Salmonella* spp. The recommended concentration of halquinol ranges from 12% to 60%, which can be associated with various dosing regimens and a withdrawal period of 10 consecutive days [2]. Researchers have demonstrated that 120 ppm of in-feed colistin led to a greater final body weight gain (BWG; 20.4 vs. 19.0 kg; *p* < 0.001) and average daily gain (ADG; 344.1 vs. 304.9 g/d; *p* < 0.05) in pigs than 120 ppm of halquinol [3]. In addition, the introduction of halquinol into the diet was not sufficient to change the composition of the microbiota or cause changes in the structure of the small intestine [4]. Nevertheless, halquinol can slow peristalsis in the gut [5]; this decelerated passage rate of digesta in the GIT may facilitate improved absorption. Beyond this, halquinol was invented to minimize the antimicrobial resistance that is common for Gram-negative bacteria, including members of the *Enterobacteriaceae* family [6,7].

Although many studies have been performed investigating the efficacy and performance of halquinol in weaned pigs, along with its elimination effect on pathogenic bacteria in GIT, to the best of our knowledge, there are limited data available comparing the effect of different concentrations of halquinol with colistin in the early weaning period in terms of digestion and microbiome modulation. In the present study, the hypothesis proposed that colistin could be replaced with an appropriate amount of halquinol supplementation, which would improve nutrient digestion, modulate the microbiome, and contribute to enhanced growth performance during the early weaning period. Therefore, the present study aimed to investigate the efficacy of colistin and halquinol, including assessments of performance, diarrhea incidence, coefficient of apparent total tract digestibility (CATTD), fecal volatile fatty acids (VFAs), and fecal microbiota.

## 2. Materials and Methods

### 2.1. Treatment Preparation

The in-feed colistin used in this study was Ascolis40^®^ (Vet products and consultant, Bangkok, Thailand), which contained 40% colistin sulfate. The in-feed halquinol used in this study was Quixalud^®^ (M & H Manufacturing Co., Ltd., Bangkok, Thailand), which contained 60% halquinol (5,7-dichloro-quinolin-8-ol).

### 2.2. Animals, Experimental Design, and Housing

At weaning, 210 piglets at 28 ± 2 days of age with an average initial weight of 6.9 kg (105 castrated males and 105 females, (Landrace × Large White) × Duroc) were randomly allocated based on the stratification of sex and weight into 5 treatment groups, and each group was divided into 6 pens (*n* = 7; *n* = 42 per treatment group). The dietary treatments included negative control (T1, basal diet), positive control (T2, basal diet + 120 ppm colistin sulfate), treatment 3 (T3, basal diet + 180 ppm halquinol), treatment 4 (T4, basal diet + 240 ppm halquinol), and treatment 5 (T5, basal diet + 360 ppm halquinol). None of the piglets had diarrhea at the time of group allocation. The pigs in each group were fed separately with treatment feed throughout the experiment. The basal diet was commercially formulated as an ABO-free diet in pellet form (HyFeed S80^®^, United feed mill, Bangkok, Thailand). The basal diet was formulated to meet the nutrient requirements of pigs based on their BW (Table 1) [8]. Pigs had ad libitum access to water and feed throughout the 10 days of the nursery feeding experiment.

The experiment was conducted in the nursery unit over the 10 d experiment period (Pikul Thong Farm, Rachaburi, Thailand). The pigs were housed in pens of 3 × 3 m^2^ (approximately 1.25 m^2^/pig; requirement 0.25 m^2^/20 kg piglet) with a concrete floor. The humidity and temperature of the pig barn were between 58% and 66% as well as 29 °C and 34 °C, respectively.

### 2.3. Field Record and Sampling Collection

Feed intake was recorded daily. Animal and feed weights were measured on the mornings of D1, D5, and D10. Prior to each feeding, any remaining feed was removed, weighed, and recorded. Experimental diets and feces were sampled on D1, D5, and D10 and held for future analysis. Feed samples were randomly collected for analysis of antibiotic composition and the homogeneity of in-feed ABO contribution. A total of 6 feces samples were collected per treatment group (1 sample/pen) on D1, D5, and D10.

Fecal samples were collected via fecal swab and frozen until processing for examination of fecal microbiota. Sub-samples were divided into 2 parts, which were stored at −80 °C until subsequent experiments. (1) Fecal samples were cultured for detection and quantification of *E. coli* [9], *Salmonella* spp. (BIO-RAD chromogenic method), total viable bacteria count [10], and coliforms. All microbiological data were analyzed and evaluated for the impact of each antibiotic on the fecal bacteria associated with PWD. (2) Fecal samples were prepared for metagenomic analysis.

### 2.4. Fecal Consistency Scores of Piglets

**Figure 1:**
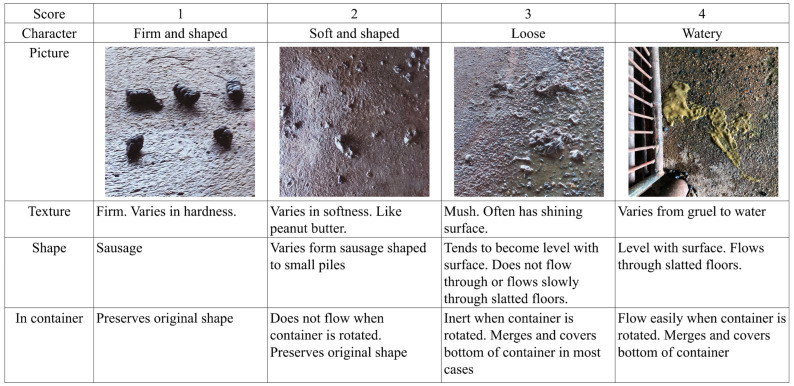
The 4 categories of fecal consistency score classification with descriptive text and pictures.

All pens were examined daily by the same person to evaluate fecal consistency according to Pedersen and Toft [11]. Scores 1 (firm and shaped) and 2 (soft and shaped) represent normal feces. Scores 3 (loose) and 4 (watery) represent diarrhea (Figure 1).

### 2.5. Nutrient Digestibility

The coefficient of apparent total tract digestibility was measured in the feces via the marker procedure. In the period from D1 to D10 (5D adaptation period + 5D collection period), 1 healthy barrow from each pen (*n* = 6) was placed in a separate metabolic cage for feces collection. After the 5-day adaptation period, feed and feces were collected and weighed. A 20 g sample was collected from each of the daily portions, which was pooled and further homogenized.

Proximate analyses of feed and feces were conducted according to AOAC [12] methods. Gross energy (GE) was determined via a bomb calorimeter (C 6000; IKA-Werke GmbH & Co., Staufen, Germany). Atomic absorption spectrophotometry (AAS) (Perkin Elmer 3110, Perkin Elmer, MA, USA) was employed to determine Cr_2_O_3_ levels using a Perkin Elmer lamp for Cr (part#303-6021 Serial H235571). The chromium standard for AAS was obtained from J.T. Baker (1000 µg/mL CAS 6449-04).

The equation for coefficient of apparent total tract digestibility of a nutrient was as follows:

$$\mathrm{CATTD}\;(\%)=\left(1-\frac{Daily\;feed\;intake\;(g\;DM/d)\times Nutrient\;concentration\;in\;diet\;(\%)}{Daily\;fecal\;output\;(g\;DM/d)\times Nutrient\;concentration\;in\;feces\;(\%)}\right)\times 100$$

### 2.6. Volatile Fatty Acids

Fecal VFA analysis was performed via gas chromatography according to the method of Kongpanna et al. [13]. Five grams of fecal matter was placed into a 15 mL centrifuge tube, followed by the addition of 2 mL of ultrapure water. The contents were vortex mixed for 30 s, incubated at 4 °C for 30 min, and subsequently centrifuged at 4 °C at 10,000× *g* for 10 min. To 1 mL of the supernatant, 0.2 mL of 25% metaphosphoric acid (*w*/*v*) was added. The solution was thoroughly mixed, incubated at 0 °C for more than 30 min, and then centrifuged at 4 °C at 10,000× *g* for 10 min. In total, 1 mL of the subsequent supernatant and 1 mL of the internal standard (0.5 g 3-methyl-n-valeric acid in 1 L of 0.15 mol/L oxalic acid) were mixed with 3 mL of distilled water. One milliliter of the supernatant was centrifuged at 10,000× *g* for 4 min at 4 °C. Then, 500 μL of the supernatant was placed into a sample bottle for measurement using gas chromatography with a flame ionization detector (GC-2010plus, Shimadzu Corporation, Kyoto, Japan).

### 2.7. 16S rRNA Sequencing of Gut Microbiota

Total DNA was extracted from 200 mg of feces per sample using a QIAamp DNA Stool Mini Kit (Qiagen, Hilden, Germany) according to the manufacturer’s protocol. The concentration and quality of genomic DNA were evaluated using a NanoDrop ND-1000 spectrophotometer (NanoDrop Technologies, Wilmington, DE, USA). The purified PCR product was assessed using 1% agarose gel electrophoresis. DNA concentrations were quantified using a Colibri Microvolume Spectrometer (Titertek Berthold, Pforzheim, Germany), and samples exhibiting OD260/280 ratios between 1.80 and 2.00 were subjected to further processing. Genomic DNA was stored for analysis at −20 °C. The V4 region of the 16S rRNA gene was amplified via polymerase chain reaction (PCR) using previously established primers [14].

### 2.8. Metagenomic Analysis

The amplicons were sequenced using the Illumina MiSeq platform according to the manufacturer’s instructions. All sequencing was performed at Macrogen Inc. (Seoul, Korea). Raw sequence data were processed using Mothur software (Version 1.45.0), and low-quality sequences were eliminated. Sequencing errors and chimeras were eliminated using UCHIME during Mothur processing [14]. The remaining high-quality sequences were categorized into operational taxonomic units (OTUs), with clustering according to an identity cutoff of 97% [15]. The sequence number was normalized via random subsampling for downstream analyses of microbial alpha diversity, such as phylogenetic information, observed OTUs, Chao1 and Shannon indices, and beta diversity (principal coordinates analysis (PCoA)).

### 2.9. Statistical Analysis

All statistical analyses were performed using the SAS ver.9.4 statistical package (SAS Institute, Cary, NC, USA). Analyses of variance (ANOVA) were performed to evaluate the impact of the treatments on piglet performance. Non-parametric tests were used because the data were not normally distributed. The Kruskal–Wallis non-parametric test was used to compare the fecal scores and microbial populations among treatments. Additionally, polynomial contrast (linear and quadratic) regressions were employed to describe the relationship between the variables and halquinol concentration. A *p*-value < 0.05 was considered statistically significant. Values outside the mean ± 3SD were treated as outliers and removed from the dataset. The fecal microbial examination and performance data are reported as mean ± SD.

A chi-squared test was used for diarrhea frequency assessment. Statistical analysis of the fecal microbial composition was performed using QIIME2. Permutational multivariate analysis of variance (PERMANOVA) was performed using QIIME2 to establish whether the profile of fecal microbiota was significantly different between the five groups and was based on UniFrac distance matrices. The alpha and beta diversities of taxonomic classification and microbial populations were analyzed using STAMP and Prism software (Prism 9.00, GraphPad Software, La Jolla, CA, USA).

## 3. Results

### 3.1. Growth Performance

The average performance results are presented (Table 2).

The BW, ADG, and FCR were not influenced by the in-feed ABO (*p* > 0.05). Meanwhile, on D10, the FCR was lower in pigs fed the T3 and T4 diets than that in pigs fed the CON, T2, and T5 diets (*p* < 0.05). Piglets receiving 240 ppm of halquinol (T4) had the lowest ADFI (*p* < 0.01). Additionally, the in-feed ABO groups had lower ADFI (*p* < 0.01) than the CON group. Therefore, these results show the reduction in FCR in piglets receiving 180 ppm (T3) and 240 ppm (T4) halquinol (*p* < 0.01). There was no significant difference between the CLT and HAL groups (T2 vs. T3–T5, *p* > 0.05) in terms of the mean values of BW, ADG, ADFI, and FCR. However, when conducting comparisons between the different HAL groups (T3 vs. T4 vs. T5), ADFI (linear and quadratic, *p* < 0.05) values were lower for piglets fed T4 compared to piglets fed T3 and T5 from days 1 to 5, 6 to 10, and during the overall period (days 1 to 10).

### 3.2. Fecal Microbial Populations

**Figure 2:**
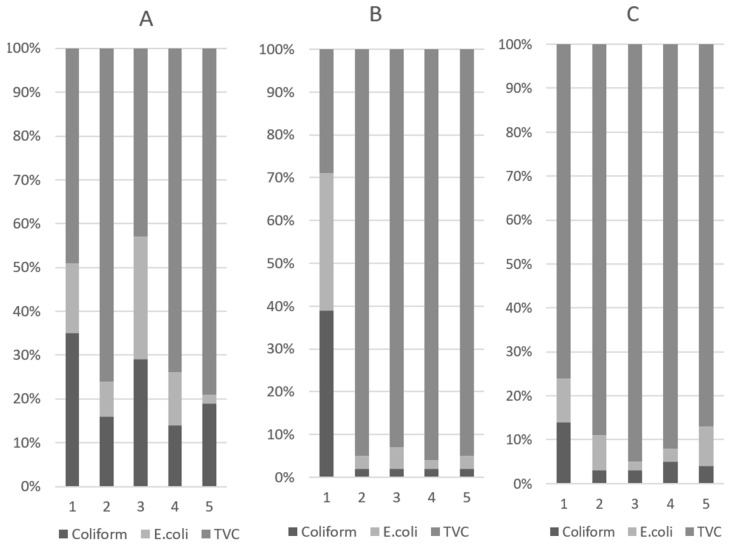
Fecal microbial proportions of coliform and *E. coli* and total viable count (TVC) in weaned pigs on D1 (**A**), D5 (**B**), and D10 (**C**). Pigs were fed a basal diet without ABO (T1), with 120 ppm of colistin (T2), or with 180, 240, or 360 ppm of halquinol (T3, T4, and T5, respectively).

The results of the analysis of fecal microbial populations and their proportions are presented in Table 3 and Figure 2, respectively. On D10, the halquinol groups had lower coliform (*p* < 0.01) and *E. coli* (*p* < 0.01) levels than the other treatment groups. In particular, adding 180 or 240 ppm of halquinol to the pigs’ diets significantly decreased the abundance of coliform (*p* < 0.01) and *E. coli* (*p* < 0.01) compared to adding colistin. This indicated that piglets fed colistin and halquinol had lower amounts of pathogenic bacteria compared with those fed a basal diet with ABOs. On D10, in pigs treated with 120 ppm (T3) of in-feed colistin, the TVC revealed variations in microbial populations (*p* < 0.001). In contrast, colistin-treated pigs also had higher coliform and *E. coli* (*p* < 0.01) proportions than pigs fed with 240 and 360 ppm halquinol.

The results for the total viable count (TVC) are also summarized in Table 3. On D5, the TVCs of the in-feed ABO groups were higher than the TVC in the control group (*p* < 0.01). On D10, the TVC of colistin was higher than that in the halquinol groups (*p* < 0.01). Furthermore, comparisons within the HAL groups (T3 vs. T4 vs. T5) showed that increasing the halquinol concentration in the diet decreased fecal coliform (linear, *p* < 0.05) and *E.coli* (linear and quadratic, *p* < 0.05) abundance and the TVC (linear, *p* < 0.05) over the experimental period (day 1 to 10).

### 3.3. Fecal Score

**Figure 3:**
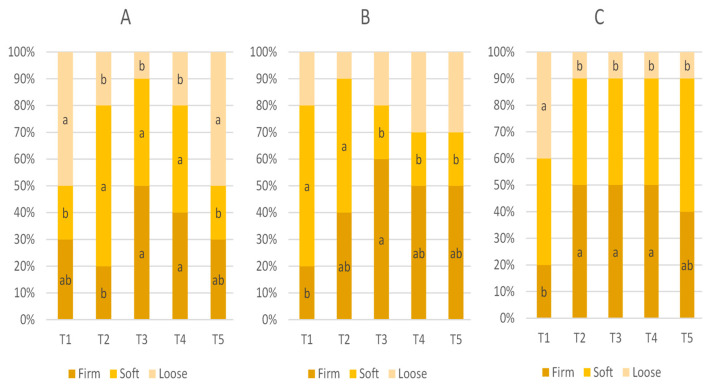
Proportions of fecal score classifications—firm, soft, and loose (represented by scores 1, 2 and 3, respectively)—for weaned pigs on D1 (**A**), D5 (**B**), and D10 (**C**). Pigs were fed a basal diet without ABO (T1), with 120 ppm of colistin (T2), or with 180, 240, or 360 ppm of halquinol (T3, T4, and T5, respectively). Letters inside the bars (a, b) indicate statistical significance (*p* < 0.05) for the fecal characteristic among the five treatments.

The effect of in-feed ABO on fecal score is presented Table 4 and Figure 3. The mean fecal score of weaned pigs on D1, D5, and D10 was not statistically significant (*p* > 0.05).

There was also no significant difference in the mean fecal score values when using CLT or HAL (T2 vs. T3–T5, *p* > 0.05); however, the scores were higher in pigs receiving the T2 and T4 diets (120 ppm of colistin and 240 ppm of halquinol, respectively).

### 3.4. Coefficient of Apparent Total Tract Digestibility

The effects of in-feed ABO on the CATTD were analyzed on D10 (Table 5). No significant differences between any parameter of the CATTD were observed among treatments, except for EED in the T4 group (*p* < 0.01). Moreover, there was also no significant difference between CLT and HAL treatments (T2 vs. T3–T5, *p* > 0.05) for the mean CATTD values of GED, DMD, OMD, CPD, EED, and CHOD.

### 3.5. Fecal Volatile Fatty Acid Production

To analyze the impacts of in-feed ABO on fecal VFA production, fecal VFA assessments were conducted on D10 (Table 6). When 240 ppm (T4) and 360 ppm (T5) of halquinol were added to the diet, the ratios of propionate and A:P changed significantly (*p* < 0.01). In addition, there was also a significant difference between CLT and HAL (T2 vs. T3–T5, *p* < 0.05) in terms of the mean propionate and A:P ratios (*p* < 0.001). Supplementation with increasing halquinol concentrations linearly decreased the A:P ratio (*p* < 0.05).

However, the total VFA, acetate, butyrate, isobutyrate, and valerate levels did not differ between the CLT and HAL groups (*p* > 0.05). In addition, there was no significant difference in the fecal concentrations of total VFAs, acetate, butyrate, isobutyrate, and valerate among the different groups (*p* > 0.05).

### 3.6. Overall Sequencing Data and Microbial Diversity of the Piglet Fecal Samples

Weaned pig fecal samples that were subjected to 16S rRNA amplicon sequencing generated 1,111,765 raw sequences (*n* = 6), ranging from 65,625 to 133,772 raw sequences per sample, after quality control and filtering steps. The Good’s coverage for all samples was always greater than 0.99. The summary of sequence counts and OTUs that passed the steps of filtering, clean-up, and normalization were grouped into 621 OTUs present in at least six samples. A total of 7 phyla, 37 families, and 83 genera were identified in the fecal samples from weaned pigs across all treatment and sampling points, with Proteobacteria, Firmicutes, Bacteroidetes, and Actinobacteria being the predominant phyla.

### 3.7. Alpha Diversity Patterns Between Treatment Groups and Sampling Times

**Figure 4:**
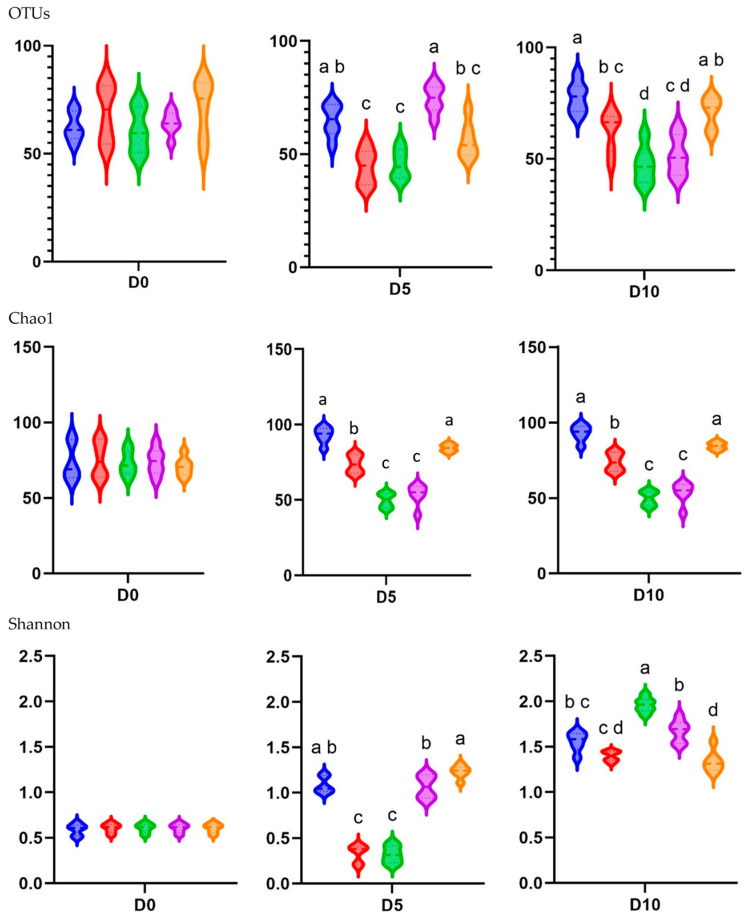
Alpha diversity represented by treatment group at the three different sampling times (D0, D5, and D10) as indicated by the OTUs observed and Chao1 and Shannon indices in the feces of weaned pigs fed a basal diet without ABO (T1, ■), with 120 ppm of colistin (T2, ■), or with 180, 240, or 360 ppm of halquinol (T3, ■; T4, ■; and T5, ■, respectively; *n* = 6). Letters inside the bars (a to d) indicate statistical significance (*p* < 0.05).

In the alpha diversity analysis, which evaluated the diversity within piglet fecal microbiota, the observed OTUs and Chao1 and Shannon indices were calculated to assess the diversity and richness of the fecal microbiota in in-feed ABO and control piglets (Figure 4).

In terms of the different treatments, the observed OTUs and Shannon alpha diversity index were higher for the T1 and T4 groups than for the T2 and T3 groups at D5 (*p* < 0.05). The Chao1 diversity index was higher for the T1 and T5 groups than for the T2, T3, and T4 groups at D5 (*p* < 0.05). The observed species, Chao1, and Shannon indices indicated significantly lower diversity on both D5 and D10 when dietary supplementation with in-feed ABOs was used (*p* < 0.05) compared with the control diet. The exception was the Shannon index on D10, where 180 ppm of halquinol (T3) led to a higher diversity than that in the CON group (T1).

**Figure 5:**
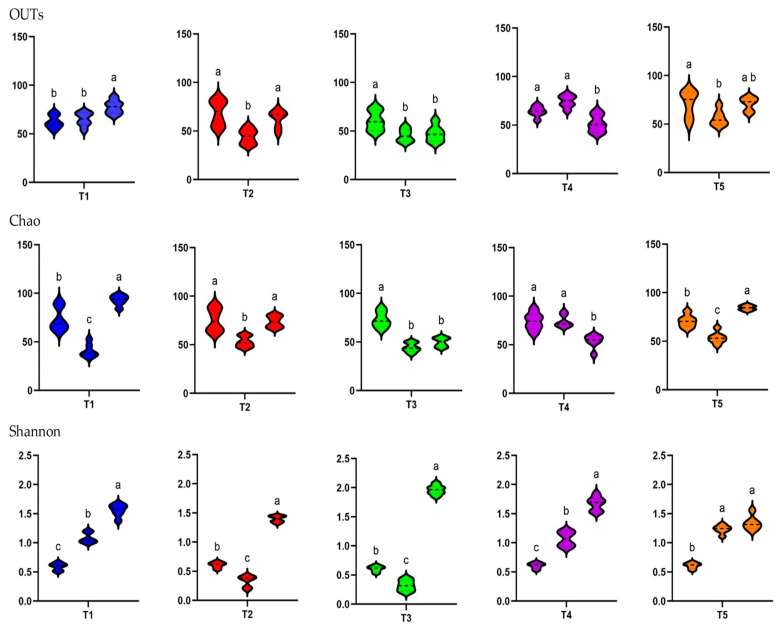
Alpha diversity in the feces of weaned pigs represented in terms of sampling time (D1, D5, and D10) per treatment group, as indicated by OTUs observed and Chao1 and Shannon indices. Pigs were fed a basal diet without ABO (T1, ■), with 120 ppm of colistin (T2, ■), or with 180, 240, or 360 ppm of halquinol (T3, ■; T4, ■; and T5, ■, respectively; *n* = 6). Letters inside the bars (a to c) indicate statistical significance (*p* < 0.05).

There was a consistent increase in alpha diversity as piglet age increased, as indicated by the observed OTUs and Shannon index. The observed OTUs, indicative of species richness, dramatically increased from day 0 to day 10 (*p* < 0.05). The Shannon index, indicative of species richness and evenness, exhibited a pronounced increase from D1 to D10, demonstrating a significant difference between these two time points (*p* < 0.05). Overall, as illustrated in Figure 5, weaning age significantly affected (*p* < 0.05) the observed species, Chao1, and Shannon indices across the treatments.

### 3.8. Beta Diversity

**Figure 6:**
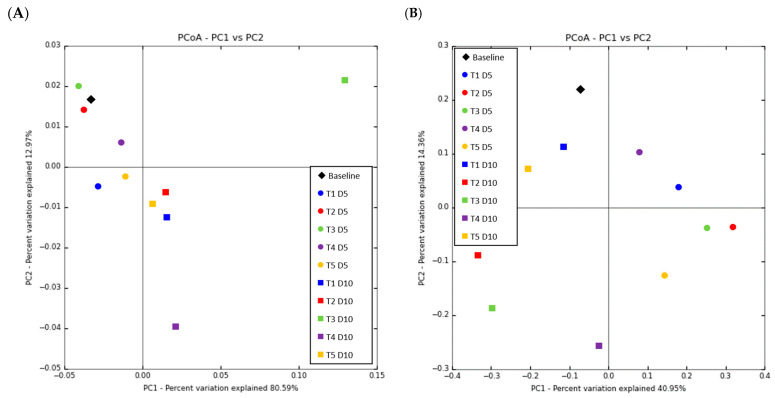
Beta diversity biplot of principal coordinate analysis (PCoA) based on the weighted (**A**) and unweighted (**B**) UniFrac distances in the feces of weaned pigs fed a basal diet without ABO (T1), with 120 ppm of colistin (T2), or with 180, 240, or 360 ppm of halquinol (T3, T4, and T5, respectively; *n* = 6).

As shown in Figure 6, a PCoA was conducted based on phylum.

Each point in the PCoA represents the microbial ecology of a piglet; points closer together have more comparable microbial ecology. PCoA based on Bray–Curtis and weighted UniFrac distances revealed clear separation of the piglets’ fecal microbiota in the T3 and T4 diets at D10; however, the other microbiota mixed together from D1 to D10. Conversely, unweighted UniFrac distances revealed a significant alteration in the fecal microbiota composition of piglets from D1 to D10 across all diets.

When PCoA was plotted on the weighted UniFrac distances, T3 and T4 exhibited a significant difference in microbiota structure, but the administration of antibiotics changed the gut microbiota structure of piglets in the in-feed ABO group. The noteworthy alterations in the fecal microbiota structure from D1 to 10 were also verified by dramatic intra-individual variations in alpha diversity from D1 to D5 and D5 to D10, which were also significantly affected by age and diet supplementation. Such inter-individual and intra-individual structural variations were also supported by weighted UniFrac distance.

### 3.9. Effects of In-Feed ABO on Bacterial Composition in Fecal Samples

**Figure 7:**
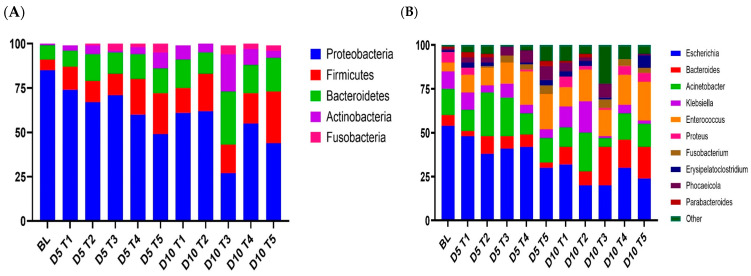
Stacked column chart showing the relative abundances of taxa in the feces of weaned pigs at the phylum (**A**) and genus (**B**) levels in the different treatment groups at different sampling times (D5 and D10). Pigs were fed a basal diet without ABO (T1), with 120 ppm of colistin (T2), or with 180, 240, or 360 ppm of halquinol (T3, T4, and T5, respectively; *n* = 6).

Across all the samples, *Proteobacteria* (59%), *Firmicutes* (17%), *Bacteroidetes* (15%), *Actinobacteria* (6%), and *Fusobacteria* (2%) were the most abundant phyla (Figure 7). At the genus level, there were nine taxa whose abundance exceeded 1% of the total sequences. *Escherichia* (33%), *Acinetobacter* (14%), *Enterococcus* (14%), *Bacteroides* (9%), and *Klebsiella* (7%) were the most predominant genera in the fecal microbiota. *Phocaeicola* (4%) and *Parabacteroides* (1%) were the two dominant genera in *Bacteroidetes*. *Erysipelatoclostridium* (2%) was the main genus in *Firmicutes*. The genus *Fusobacterium*, classified under *Fusobacteria*, constituted 2% of the total sequences.

The phylum-level composition of the gut microbiota in each sample revealed significant variations in bacterial community structure among piglets of different ages (D1 vs. D5 vs. D10). *Proteobacteria*, the most prevalent phylum, had notable reductions in relative abundance on day 5 compared to day 10, but *Bacteroides* demonstrated considerable increases in relative abundance. The phylum-level results showed that weaning on both day 5 and 10 did not affect (*p* > 0.05) the abundance of *Firmicutes*, *Bacteroidetes*, and *Actinobacteria*. Other phyla (*Spirochaetes*, *Fusobacteria*, and *Synergistetes*) were found at minimal relative abundances, with no significant differences observed (*p* > 0.05).

At the genus level, there was significant heterogeneity in the gut microbiota composition dependent on piglet age (D1 vs. D5 vs. D10).

*Escherichia*, the most prevalent phylum, had a notable decline in relative abundance on day 5 compared to day 10, but *Bacteroides* demonstrated a large increase in relative abundance. The relative abundances of *Acinetobacter*, *Klebsiella*, *Enterococcus*, *Proteus*, *Fusobacterium*, and *Erysipelatoclostridium*, representing the main phylum in the analysis, did not vary significantly with age.

We also conducted a genus-level comparison of the microbial population by dietary treatment. In-feed ABO supplementation led to a decrease in *Escherichia* abundance compared to the CON group on D5 and a significant decrease in *Escherichia* on D10 (*p* < 0.01). *Bacteroidetes* was present at significantly higher levels in the Hal group (16.02 to 20.94%; *p* < 0.01) than in the CON and HAL groups (9.86 and 7.82%, respectively), despite the fact that the CON and HAL groups did not differ much. The relative abundance of *Acinetobacter* in the COL group on D5 and D10 (24.04 and 21.01%, respectively) was significantly higher than that in the CON and HAL group (*p* < 0.05). The relative abundance of *Klebsiella* was significantly higher in the CLT group on D10 (17.68%), while it was significantly lower in the HAL group on D10 (2.74%; *p* < 0.01). *Enterococcus* was present at significantly higher levels in the CLT and HAL groups (17.93% and 15.05 to 21.21%; *p* < 0.01) than in the CON group (11.04%) on D10.

### 3.10. Correlation Analysis

**Figure 8:**
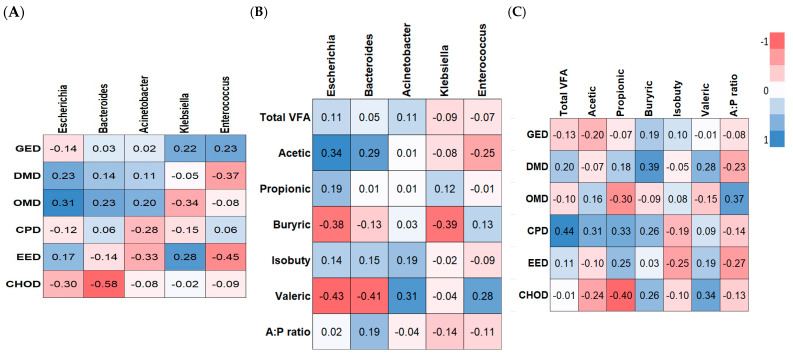
Heatmap of the Pearson’s correlations between the significantly modified microbiota and the ATTD (**A**), VFAs (**B**), and ATTD vs. VFAs (**C**) parameters of weaned pigs.

The possible relationships between the changes in the composition of fecal microbiota and ATTD (Figure 8A), VFAs (Figure 8B), and ATTD vs. VFAs (Figure 8C) were assessed using Pearson’s correlation analysis. The genus Bacteroides was negatively correlated with CHOD levels (R = −0.58, *p* < 0.01). The genus *Enterococcus* was negatively correlated with EED (R = −0.45, *p* < 0.05). The genera Acinetobacter, Klebsiella, and Enterococcus trended toward being negatively correlated with EED, OMD, and DMD, respectively (R = −0.30, *p* < 0.10). Only Escherichia trended toward being positively correlated with OMD (R = 0.31, *p* < 0.10).

The genus *Escherichia* trended toward being positively correlated with levels of acetic acid (R = 0.36, *p* < 0.10), while it was significantly negatively correlated with the levels of butyric and valeric acids (R = 0.38 and R = 0.43, *p* < 0.05). The genus *Bacteroides* was negatively correlated with the levels of valeric acid (R = 0.41, *p* < 0.05). The genus *Klebsiella* was negatively correlated with butyric acid levels (R = 0.39, *p* < 0.05). The genus *Acinetobacter* trended toward being positively correlated with the levels of valeric acid (R = 0.31, *p* < 0.10). The concentration of propionic acid was negatively correlated with CHOD levels (R = 0.40, *p* < 0.05).

## 4. Discussion

The impact of the substitution of in-feed ABO in swine production was evaluated. According to our hypothesis, these antibiotics can influence the commensal microbial community and could promote a healthy GIT in the early weaning period. This study examined the composition and functional aspects of the fecal microbiota in the weaning period via fecal sample analysis.

Our findings indicate that in-feed ABOs did not positively influence BWG and ADG but significantly affected ADFI and FCR, especially in the group receiving 240 ppm halquinol (T4). This was verified by Barbosa et al. [16] and Grecco et al. [17], whose experiments used halquinol levels ranging from 60 to 200 ppm. Thus, in-feed ABO supplementation could not only control PWD but also optimize growth performance. Comparative studies on the use of in-feed ABO, such tilmicosin, amoxicillin, and doxycycline, in the weaning period concluded that growth performance was not influenced by these in-feed ABOs, despite the fact that pigs receiving ABO supplementation reached slaughter 2 kg heavier than those that did not [18]. These results support our hypothesis that the inclusion of 240 to 360 ppm halquinol in a pig’s diet may contribute to a balanced microbial composition, a healthy gastrointestinal environment, and the preservation of intestinal structure, which contributes to growth in later development stages. Tummarak et al. [3] added 120 ppm of halquinol to pig diets, which was insufficient to improve piglet performance metrics compared to 120 ppm of colistin. The discrepancy could be explained by the mechanisms of colistin. Colistin targets Gram-negative bacteria, including *Salmonella*, *Escherichia coli*, *Klebsiella*, and *Pseudomonas*. It binds to phospholipids and lipopolysaccharides, disrupting bacterial cell membranes and inhibiting the release of endotoxins from dead bacteria.

Based on our review of the literature, concentrations of 180 to 360 ppm of halquinol were selected and validated for our experiment as in-feed ABO replacements to improve the efficiency of feed utilization, reduce harmful microorganisms, and promote beneficial microbial growth in the GIT without negative consequences for the piglet [3,16,17].

This study showed that piglets fed 240 ppm halquinol had significantly lower ADFI during the first 10 days after weaning, resulting in not only lower FCR but also lower diarrhea incidence. The association between pathogenic bacteria and ADFI could be explained by the intestinal environments of newly weaned pigs being susceptible to invasion by pathogenic bacteria, which impairs the digestive system. The consequence of this is damage to the GIT, which affects the pigs’ ability to digest and absorb nutrients; therefore, their food intake might need to increase significantly just to reach their minimum nutritional requirements. Additionally, undigested materials may be available for utilization by, e.g., enterotoxigenic *E. coli*, resulting in infectious diarrhea [19]. Therefore, the low ADFI in piglets fed 240 ppm halquinol may actually indicate the effectiveness of the in-feed ABO in enhancing nutrient utilization, given the low concentration of undigested and unabsorbed contents in the intestinal lumen [20]. Moreover, the presence of quadratic contrast affected piglets’ ADFI over the entire period. This may explain why high levels of halquinol supplementation (T5; 360 ppm of halquinol) were found to suppress growth performance and reduce antimicrobial effectiveness.

During weaning, the intestinal flora underwent a dramatic change in diversity and composition, and in-feed ABO supplementation may have changed the number of bacteria in each species [20,21]. In the present study, the fecal *E.coli* levels and TVC were significantly decreased in halquinol groups compared to the colistin group at D10 (T2 vs. T3 to T5). This suggests that halquinol has the potential to significantly change the microbial structure of the host’s gut by boosting the abundance of beneficial bacteria and lowering the prevalence of harmful bacteria [22].

Surprisingly, 240 ppm of halquinol can enhance fat digestion, leading to energy storage. This treatment, with its associated enhanced feed efficiency (ADFI and FCR) and reduced ADFI, FCR, and passage of food in the gut, may help enhance absorption in the first three weeks after weaning [5]. Although the biological relevance of lingual lipase diminishes with pig age, lingual and pancreatic lipase activities can be comparable to or higher than stomach lipases in neonatal piglets [23]. The interaction between halquinol and fat digestion is thought to enhance digestion capacity, which subsequently leads to a deceleration of the passage rate process. This enhances the digestion of lipid fat, which may require a longer duration for digestion compared to other nutrients.

Subsequently, we determined that the propionate concentrations in feces were significantly higher in the halquinol group than in the control and colistin groups. The results indicated that undigested CHO could be a substance the allows beneficial microbes to produce propionate, as confirmed in the correlation study. Propionate is known to regulate cellular function and the immune response [24]. Collectively, supplementation with halquinol increased propionic acid levels and fat digestion, both of which are related to improved feed efficiency. Although improvements in feed efficiency, reduced diarrhea occurrence, and increased VFA production were observed in animals that were fed halquinol, none of the dietary treatments positively affected piglet performance. Halquinol’s mechanisms of action are described by Hall et al. [2]; it primarily functions as an antimicrobial, anti-inflammatory, and gut health modulator through stabilizing gut pH, disrupting bacterial DNA and enzyme systems, reducing pathogenic bacteria, and enhancing nutrient absorption by maintaining gut integrity.

The diversity and richness indices are frequently used to measure the gut microbiota’s functional stability [20,21]. Our study demonstrated that the abundance and diversity (OTUs and Shannon index) of fecal microbiota increase as weaned piglets age. Our findings are in accordance with Feehan et al. [25], who asserted that alpha diversity undergoes substantial alterations during this transition period. The present study revealed that the alpha diversity of the fecal bacteriome remained stable from day 0 to day 5 and subsequently grew from day 5 to day 10. Additionally, in-feed ABOs can diminish alpha diversity and induce alterations in gut microbiota due to their broad-spectrum activity and capacity to eliminate or inhibit the proliferation of both pathogenic and beneficial microorganisms [26].

Pig fed in-feed colistin and halquinol had decreased fecal microbiota diversity (OTUs and Chao1). This result was consistent the findings of from Abeles et al. [27] and Chen et al. [28], who found that alpha diversity was reduced with in-feed ABO supplementation. Thus, the less diverse and more dynamic microbial community in the guts of pigs in the early weaned period may be more vulnerable than that in weaning pigs and thus can be easily influenced by environmental factors, for instance, the antibiotic exposure in the current study. In fact, the authors of [29] found that more efficient microbiomes are prone to reduced diversity and result in a smaller variation in metabolites. Nevertheless, within that diminished pool of metabolites, there could be a greater concentration of biologically valuable metabolites that are more readily accessible for utilization by the host.

In contrast, increases in microbial diversity are often positively associated with the resilience of microflora against disease invasion. Similarly, the improvement of alpha and beta diversity indicated that the relative abundance of beneficial bacteria had increased [28]. The contribution of diversity was mainly reflected in the modification, structure, and abundance of the microbiota and the maintenance of intestinal homeostasis. Furthermore, the antibiotics significantly influenced microbial alterations in the foregut but not in the hindgut. Additionally, early exposure to antibiotics does not substantially alter the intestinal ecological environment.

The alpha diversity of the fecal microbiome appears to follow a linear trend, likely due to the strong impact of halquinol toward restoring diversity. Overall, piglets receiving 360 ppm halquinol had a lower fecal bacterial biodiversity than the other groups. Supplementation with 180 and 240 ppm halquinol led to significant differences in fecal microbiome composition, reflecting a richness in alpha diversity. Similarly, it has been shown that pigs fed antibiotics have enhanced expression of functional genes that affect their microbiome and are associated with energy production and feed efficiency, suggesting that consumption of antibiotics leads to a more effective utilization of energy from feed [30]. Moreover, the alpha diversity results, except for the Shannon index, indicate there are age-related differences, with diversity gradually increasing in the piglets as they age from 0 to 10 days after weaning. Alternatively, Huaman et al. [31] demonstrated that weaned pigs receiving 200 ppm of halquinol had lower Chao1 alpha diversity and observed OTUs than a group receiving synbiotics. Living microorganisms, such as probiotics or synbiotics, often increase microbial richness and stability, which increases alpha diversity in weanling pigs, while biological and environmental factors can affect the outcome [31].

Beta diversity analysis can corroborate this finding. Halquinol influenced the composition of fecal microbiota in weaned pigs, altering the distance between control group and in-feed ABO group samples in PCoA analysis. However, the beta diversity between all three age points was different, indicating that both dietary treatment and age affected it. Although there was a significant increase in Shannon diversity on D10, taxonomic changes occurred and were the primary driver of diversity changes.

Furthermore, altered microbial composition has been associated with the production and composition of VFAs in the colon. The current study revealed that the fecal concentrations of VFAs, specifically propionic acid, dramatically increased in piglets whose diets were supplemented with 240 or 360 ppm halquinol; propionic acid is an essential metabolite of gut microbiota and may promote energy homeostasis and alleviate inflammation and metabolic disorders in the colon [32]. In-feed ABO consumption was not the only factor influencing microbiota diversity; the PWD caused by ETEC also affected microbiota imbalances via the increased growth of species of pathogenic bacteria [33].

Our findings reveal that *Proteobacteria* (59%), *Firmicutes* (17%), and *Bacteroidetes* (15%) were the predominant phyla among the five groups of weaned piglets, supporting earlier studies in pigs [33,34]; however, the proportion of *Proteobacteria* was indicative of the high frequency of PWD. Elevated levels of *Helicobacteraceae*, a family within *Proteobacteria*, have been documented to weaken the protective mucous layer in the gut, whereas *Prevotellaceae*, a member of the *Bacteroidetes* phylum, has been linked to fortifying the intestinal mucosa of healthy pigs fed plant-based diets [35,36]. Moreover, the high percentage of rice bran in the diet might be a reason for the predominance of *Proteobacteria*, as this ingredient contains phytic acid and a trypsin inhibitor [37], which negatively affect the digestive system.

The current study shows that in the early weaning period (first 5 days post-weaning), the fecal microbiota structures were similar among the five dietary treatments, except that the relative abundance of *Enterococcus* was higher in pigs fed 240 to 360 ppm halquinol than in pigs in the other groups. In particular, halquinol had no effect on the bacteria that are the most common in feces. Compared to the control groups, the types of bacteria that were most common in the halquinol groups were *Escherichia* (24%), *Bacteroides* (18%), and *Enterococcus* (17%). The rise in beneficial *Enterococcus* bacteria belonging to the phylum *Firmicutes* could be positively correlated with metabolites associated with inducing oxidative stress [38].

A pig’s gut microbiota colonization begins at birth but continues to develop during the weaning process [39]. Even though *E. coli* is among the first bacteria to colonize in piglets’ intestines [40], it is still dominant after weaning. The presence of ETEC infection or heightened levels of *E. coli* during the post-weaning phase may influence the gut microbiota. ETEC K88 infection decreased microbial diversity and suppressed *Bacteroidetes* and *Firmicutes* proliferation in the feces and jejunum of weaned pigs [41]. The proliferation of *Firmicutes* and *Bacteroidetes* in pigs’ gastrointestinal tracts contributes to the animal’s general health in numerous ways. In this study, pigs fed with 360 ppm halquinol (T5) had higher abundances of *Enterococcus* and *Erysipelatoclostridium*, which belong to phylum *Firmicutes*, as well as higher *Bacteroides* abundance compared to control and colistin diets. *Firmicutes* are present in the GIT of pigs and are responsible for the body’s energy balance and utilization of carbohydrates in the gut [42]. *Bacteroides* are crucial for health, as they generate butyrate, which activates T cell-mediated immune responses that minimize pathogenic bacteria’s colonization of the gastrointestinal tract [43].

The highest abundances of *Bacteroidetes* were mostly found in pigs fed with halquinol. *Bacteroides* and *Parabacteroides*, which are present in the early stages of life, have been documented to synthesize gamma-aminobutyric acid (GABA), which is linked to growth [44]. Moreover, post-weaning diarrhea has been linked to the low abundance of the genus *Bacteroides*, which is also thought to be a mucin glycan degrader [45]. Microbial glycan degradation results in the generation of metabolites that help to modulate mucosal health.

The phylum *Bacteroidetes* has a unique ability to digest carbohydrates, including simple and complex carbohydrates as well as polysaccharides [46]. Conversely, an elevation in the prevalence of *Bacteroides* is typically observed in cases of ulcerative colitis, colorectal cancer, and functional gastrointestinal problems [47].

Furthermore, it was observed that *Fusobacteria* were only present in pigs fed diets supplemented with halquinol. This observation may be attributed to a decrease in the proportion of beneficial bacteria and an increase in the prevalence of harmful bacteria. Generally, based on this study’s control group, the abundance of *Fusobacterium* decreased by more than 70% from D1 to D10. As a result, newly weaned pigs’ gut microbial composition may be readily disrupted by a decrease in diversity, making pigs more susceptible to pathogenic microbes. On the other hand, halquinol fails to modulate and control the invasion of *Fusobacterium*. Infections with *Fusobacterium* spp. have been linked to a wide range of pathologies. Relevance networking also revealed that *Fusobacterium* was negatively associated with α-diversity, as shown previously [48,49]. *Fusobacterium* is emerging as a pathogen that is associated with gastrointestinal diseases such as ulcerative colitis [50].

*Enterococcus faecium* was found in both the control group and the in-feed ABO groups. This species is indicative of normal gut flora and is the most common type of bacteria in piglets when they are young. A high abundance of the *Enterococcus* genera may be essential for post-weaning piglets to digest plant-based diets. *Enterococcus* can form biofilm microcolonies throughout the gastrointestinal tract, increase immunoreactivity, and reduce instances of diarrhea [51]. Researchers have conducted numerous studies to evaluate the probiotic characteristics of *Enterococcus* strains, primarily *E. faecium*. Feeding pigs with the probiotic *Enterococcus* was found to reduce intestinal pathogens, improve the gut microbiota balance, and improve the growth and feed conversion of piglets [52]. Thus, in the current study, the lack of relative abundance data for various genera in the feces of piglets may be explained by the short experiment duration, which might have been insufficient to fully impact the microbiota diversity.

Finally, our results indicate that excessive *Escherichia* abundance and insufficient *Bacteroides* abundance serve as indicators of digestive issues in the early weaning period (D0 to D5), because changes in their abundance have been correlated with impaired nutrient digestion and various disorders resulting from PWD [53]. At weaning, the intestinal microbiota is unstable and the animals’ immune and digestive systems are not fully developed, which makes the piglets highly susceptible to PWD [39]. The halquinol-mediated alterations in beneficial microbiota may be attributed to fat utilization and VFA regulation, which might be key modulators in the maintenance of intestinal homeostasis after weaning.

## 5. Conclusions

In conclusion, 240 ppm halquinol (Quixalud^®^) is a potentially viable alternative to in-feed colistin for the early weaning period (the first 10 days after weaning) in terms of its ability to improve feed efficiency and fat digestion, control the proliferation of pathogenic bacteria, augment propionate-producing bacteria, and modulate beneficial microbiota. Consequently, a balanced ratio of *Escherichia* and *Bacteroides*, an advantageous gastrointestinal environment, and the maintenance of intestinal morphology are established during the early weaning period, resulting in healthier piglets in the later stages of growth.

## Figures and Tables

**Table 1:** Ingredient (% as feed) and chemical composition of the feed used in the current study.

Ingredient, %	
Corn	20.17
Soybean meal	27.36
Rice bran	32.76
Whey powder	8.00
Lactose	4.00
Soy peptide	4.74
Mono-dicalcium phosphate	1.14
Limestone	0.95
L-Lysine-HCL, 78%	0.20
DL-Methionine, 99%	0.04
L-Threonine, 99%	0.09
Salt	0.30
^1^ Vitamin/mineral premix	0.25
Chemical composition ^2^	
ME (Mcal/kg)	3450
Dry matter (%)	90.23
Crude protein (%)	22.69
Crude fat (%)	5.07
Crude fiber (%)	8.06
Total lysine (%)	1.35
Total methionine (%)	0.35
Total threonine (%)	0.89
Calcium (%)	0.80
Total phosphorus (%)	0.65

^1^ Supplied 2.40 IU vitamin A, 0.48 IU vitamin D, 4000 IU vitamin E, 0.72 g vitamin K, 0.069 g vitamin B1, 1.04 g vitamin B2, 0.12 g vitamin B6, 0.006 g vitamin B12, 7.20 g nicotinic acid, 2.72 g pantothenic acid, 34.88 g Cu as copper sulfate, 20 g Fe as ferrous sulfate, 0.23 g I as calcium iodate, 13.64 g Mn as manganese sulfate, 24 g Zn as zinc sulfate, and 0.02 g Se as sodium selenite. ^2^ Calculated values.

**Table 2:** Body weight (BW), average daily gain (ADG), average daily feed intake (ADFI), and feed conversion ratio (FCR) of weaned pigs on D1, D5, and D10. Pigs were fed a basal diet without ABO (T1), with 120 ppm of colistin (T2), or with 180, 240, or 360 ppm of halquinol (T3, T4, and T5, respectively).

		CON	CLT	HAL	HAL	HAL	HAL	*p*-Value		
		T1	T2	T3	T4	T5	T3–T5	ANOVA ^1^	CLT vs. HAL	Contrast ^2^
BW, kg	D1	6.90 ± 0.90	6.90 ± 0.86	6.92 ± 0.82	6.83 ± 1.31	6.95 ± 1.31	6.90 ± 1.15	0.999	0.997	-
	D5	9.20 ± 1.20	9.20 ± 1.14	9.23 ± 1.10	9.10 ± 1.75	9.26 ± 1.74	9.20 ± 1.54	0.988	0.789	-
	D10	12.06 ± 1.34	12.00 ± 1.41	11.95 ± 1.13	11.96 ± 1.39	11.99 ± 1.73	11.97 ± 1.42	0.807	0.571	-
ADG, g/day	D1–D5	329 ± 43	329 ± 41	330 ± 39	325 ± 62	331 ± 62	329 ± 55	0.985	0.759	-
	D6–D10	408 ± 203	400 ± 219	389 ± 142	408 ± 163	390 ± 210	395 ± 171	0.577	0.434	-
	D1–D10	368 ± 93	364 ± 101	359 ± 65	366 ± 68	360 ± 96	362 ± 76	0.839	0.620	-
ADFI, g/pig/day	D1–D5	714 ± 38 ^a^	677 ± 27 ^c^	684 ± 68 ^bc^	628 ± 37 ^d^	696 ± 48 ^b^	669 ± 60	0.001	0.551	Q
	D6–D10	825 ± 53 ^a^	783 ± 36 ^b^	745 ± 54 ^c^	726 ± 64 ^d^	795 ± 54 ^b^	755 ± 64	0.001	0.057	Q
	D1–D10	769 ± 45 ^a^	730 ± 31 ^c^	715 ± 61 ^d^	677 ± 51 ^e^	746 ± 51 ^b^	712 ± 60	0.001	0.163	Q
FCR	D1–D5	1.38 ± 0.46	1.34 ± 0.50	1.45 ± 0.40	1.36 ± 0.41	1.38 ± 0.42	1.40 ± 0.41	0.934	0.611	-
	D6–D10	1.64 ± 0.33	1.58 ± 0.43	1.56 ± 0.39	1.41 ± 0.41	1.68 ± 0.48	1.55 ± 0.43	0.399	0.648	-
	D1–D10	1.72 ± 0.35 ^a^	1.54 ± 0.29 ^ab^	1.49 ± 0.29 ^b^	1.44 ± 0.35 ^b^	1.63 ± 0.35 ^ab^	1.52 ± 0.34	0.044	0.782	-

^a,b,c,d,e^ Means ±  SD with superscript letters indicate a difference of *p*  <  0.05; ^1^ CON (T1, basal diet), CLT (T2, basal diet + 120 ppm colistin sulfate), HAL (T3, basal diet + 180 ppm halquinol; T4, basal diet + 240 ppm halquinol; T5, basal diet + 360 ppm halquinol). ^2^ Orthogonal polynomial contrasts: Q = quadratic regression.

**Table 3:** Fecal microbial populations in units of Log10 in weaned pigs on D1, D5, and D10. Pigs were fed a basal diet without ABO (T1), with 120 ppm of colistin (T2), or with 180, 240, or 360 ppm of halquinol (T3, T4, and T5, respectively).

		CON	CLT	HAL	HAL	HAL	HAL	*p*-Value		
		T1	T2	T3	T4	T5	T3–T5	ANOVA ^1^	CLT vs. HAL	Contrast ^2^
Coliforms, cfu/g	D1	6.95 ± 0.52 ^ab^	7.55 ± 0.19 ^a^	6.64 ± 0.27 ^b^	6.58 ± 0.18 ^b^	6.40 ± 1.10 ^b^	6.54 ± 0.61	0.002	0.006	-
	D5	6.12 ± 0.33	5.95 ± 0.19	5.81 ± 0.30	6.29 ± 0.83	5.82 ± 0.79	5.97 ± 0.66	0.771	0.942	-
	D10	6.03 ± 0.74 ^a^	5.59 ± 0.49 ^ab^	4.64 ± 0.23 ^c^	4.89 ± 0.35 ^bc^	5.64 ± 0.22 ^a^	5.06 ± 0.51	0.005	0.091	L
*E.coli*, cfu/g	D1	6.70 ± 0.26	7.26 ± 0.51	6.71 ± 0.17	6.51 ± 0.29	6.15 ± 0.73	6.46 ± 0.49	0.075	0.039	-
	D5	6.08 ± 0.24	6.06 ± 0.42	5.62 ± 1.56	6.00 ± 0.65	6.13 ± 0.57	5.91 ± 0.96	0.897	0.689	-
	D10	5.77 ± 0.58 ^a^	6.13 ± 0.49 ^a^	4.57 ± 0.18 ^b^	4.79 ± 0.27 ^b^	5.91 ± 0.34 ^a^	5.09 ± 0.66	0.001	0.012	-
TVC ^3^, cfu/g	D1	7.59 ± 0.17 ^c^	8.51 ± 0.41 ^a^	7.29 ± 0.18 ^c^	7.50 ± 0.23 ^c^	8.12 ± 0.17 ^b^	7.64 ± 0.41	0.001	0.002	L
	D5	6.63 ± 0.17 ^c^	8.58 ± 0.26 ^b^	8.70 ± 0.13 ^ab^	8.92 ± 0.21 ^a^	8.46 ± 0.27 ^b^	8.69 ± 0.28	0.001	0.497	Q
	D10	7.13 ± 0.61 ^a^	7.58 ± 0.14 ^a^	6.48 ± 0.32 ^b^	6.49 ± 0.26 ^b^	7.15 ± 0.31 ^a^	6.71 ± 0.42	0.006	0.001	L
Salmonella	D1	<10	<10	<10	<10	<10				
	D5	<10	<10	<10	<10	<10				
	D10	<10	<10	<10	<10	<10				

^a,b,c^ Means ± SD with superscript letters indicate differences of *p*  <  0.05; ^1^ CON (T1, basal diet), CLT (T2, basal diet + 120 ppm colistin sulfate), HAL (T3, basal diet + 180 ppm halquinol; T4, basal diet + 240 ppm halquinol; T5, basal diet + 360 ppm halquinol). ^2^ Orthogonal polynomial contrasts: L = linear and Q = quadratic regression. ^3^ TVC = Total viable count.

**Table 4:** Mean fecal scores of weaned pigs on D1, D5, and D10. Pigs were fed a basal diet without ABO (T1), with 120 ppm of colistin (T2), or with 180, 240, or 360 ppm of halquinol (T3, T4, and T5, respectively).

		CON	CLT	HAL	HAL	HAL	HAL	*p*-Value		
		T1	T2	T3	T4	T5	T3–T5	ANOVA ^1^	CLT vs. HAL	Contrast
Fecal score	D1	2.20 ± 0.92	2.00 ± 0.67	1.60 ± 0.70	1.80 ± 0.79	2.20 ± 0.92	1.87 ± 0.82	0.393	0.642	-
	D5	2.00 ± 0.67	1.70 ± 0.67	1.60 ± 0.84	1.80 ± 0.92	1.80 ± 0.92	1.73 ± 0.87	0.854	0.914	-
	D10	2.20 ± 0.79	1.60 ± 0.70	1.60 ± 0.70	1.60 ± 0.70	1.70 ± 0.67	1.63 ± 0.67	0.271	0.897	-

Means ±  SD with superscripts indicate differences of p  <  0.05; ^1^ CON (T1, basal diet), CLT (T2, basal diet + 120 ppm colistin sulfate), HAL (T3, basal diet + 180 ppm halquinol; T4, basal diet + 240 ppm halquinol; T5, basal diet + 360 ppm halquinol).

**Table 5:** Coefficient of apparent total tract digestibility (CATTD) of weaned pigs on D10. Pigs were fed a basal diet without ABO (T1), with 120 ppm of colistin (T2), or with 180, 240, or 360 ppm of halquinol (T3, T4, and T5, respectively).

		CON	CLT	HAL	HAL	HAL	HAL	*p*-Value		
	D10	T1	T2	T3	T4	T5	T3–T5	ANOVA ^1^	CLT vs. HAL	Contrast ^2^
GED ^3^	%	81.68 ± 3.96	81.61 ± 4.34	80.69 ± 3.60	82.01 ± 2.71	83.21 ± 4.29	81.97 ± 3.42	0.917	0.818	-
DMD	%	80.68 ± 1.52	82.36 ± 2.13	85.44 ± 3.60	83.76 ± 4.26	84.71 ± 3.14	84.64 ± 3.42	0.192	0.743	-
OMD	%	87.93 ± 3.38	85.61 ± 4.43	83.94 ± 3.01	86.26 ± 5.31	82.71 ± 3.14	84.31 ± 3.90	0.471	0.627	-
CPD	%	79.68 ± 5.61	85.61 ± 3.77	82.94 ± 7.65	82.51 ± 1.31	83.96 ± 5.44	83.14 ± 4.99	0.490	0.317	-
EED	%	70.93 ± 3.13 ^c^	74.97 ± 1.53 ^bc^	75.19 ± 3.80 ^bc^	84.27 ± 2.48 ^a^	77.71 ± 5.69 ^b^	78.59 ± 5.47	0.006	0.168	Q
CHOD	%	91.43 ± 3.12	89.11 ± 2.97	89.94 ± 4.05	88.76 ± 3.72	89.21 ± 2.96	89.31 ± 3.30	0.830	0.857	-

^a,b,c^ Means ±  SD with superscript letters indicate differences of p  <  0.05; ^1^ CON (T1, basal diet), CLT (T2, basal diet + 120 ppm colistin sulfate), HAL (T3, basal diet + 180 ppm halquinol; T4, basal diet + 240 ppm halquinol; T5, basal diet + 360 ppm halquinol). ^2^ Orthogonal polynomial contrasts: Q = quadratic regression. ^3^ GED = gross energy digestibility, DMD = dry matter digestibility, OMD = organic matter digestibility, CPD = crude protein digestibility, EED = ether extract digestibility, and CHOD = carbohydrate (starch) digestibility.

**Table 6:** Fecal volatile fatty acids (VFAs) in weaned pigs on D10. Pigs were fed a basal diet without ABO (T1), with 120 ppm of colistin (T2), or with 180, 240, or 360 ppm of halquinol (T3, T4, and T5, respectively).

		CON	CLT	HAL	HAL	HAL	HAL	*p*-value		
	D10	T1	T2	T3	T4	T5	T3–T5	ANOVA ^1^	CLT vs. HAL	Contrast ^2^
Total VFAs	mmol/g feces	113.61 ± 11.88	114.02 ± 4.50	121.61 ± 1.34	117.32 ± 6.17	119.18 ± 6.84	119.37 ± 5.20	0.470	0.088	-
Acetate	mmol/g feces	66.48 ± 6.68	70.28 ± 4.26	71.65 ± 1.92	67.23 ± 5.39	66.08 ± 3.59	68.32 ± 4.33	0.378	0.444	-
Propionate	mmol/g feces	24.10 ± 3.23 ^bc^	22.73 ± 1.84 ^c^	26.70 ± 1.97 ^ab^	28.55 ± 2.98 ^a^	28.85 ± 2.11 ^a^	28.03 ± 2.38	0.011	0.001	-
Butyrate	mmol/g feces	7.03 ± 1.51	6.43 ± 2.31	6.65 ± 1.88	6.30 ± 0.91	7.00 ± 1.19	6.65 ± 1.29	0.954	0.807	-
Isobutyrate	mmol/g feces	0.56 ± 0.22	0.62 ± 0.17	0.51 ± 0.20	0.54 ± 0.22	0.70 ± 0.14	0.58 ± 0.19	0.655	0.759	-
Valerate	mmol/g feces	15.45 ± 1.63	13.98 ± 1.88	16.10 ± 1.89	14.70 ± 2.89	16.55 ± 2.34	15.78 ± 2.33	0.480	0.184	-
A:P ratio		2.78 ± 0.25 ^ab^	3.11 ± 0.36 ^a^	2.70 ± 0.22 ^bc^	2.36 ± 0.17 ^cd^	2.29 ± 0.09 ^d^	2.45 ± 0.24	0.001	0.001	L

^a,b,c,d^ Means ± SD with superscript letters indicate differences of p  <  0.05; ^1^ CON (T1, basal diet), CLT (T2, basal diet + 120 ppm colistin sulfate), HAL (T3, basal diet + 180 ppm halquinol; T4, basal diet + 240 ppm halquinol; T5, basal diet + 360 ppm halquinol). ^2^ Orthogonal polynomial contrasts: L = linear regression.

## Data Availability

The data that support the findings of this study are available on re-quest from the corresponding author.

## Abbreviations

The following abbreviations are used in this manuscript:

ABO	Antibiotics
ADG	Average daily gain
BWG	Body weight gain
CATTD	Coefficient of apparent total tract digestibility
CHO	Carbohydrate
CLT	Colistin group
CON	Control group
CP	Crude protein
DLD	Department of Livestock Development
DM	Dry matter
EE	Ether extract
FCR	Feed conversion ratio
GE	Gross energy
GIT	Gastrointestinal tract
HAL	Halquinol group
OM	Organic matter
SCFA	Short-chain fatty acid
ppm	Parts per million
PWD	Post-weaning diarrhea
VFA	Volatile fatty acid

## References

[1] Fairbrother J.M., Nadeau E., Gyles C.L. (2005). *Escherichia coli* in postweaning diarrhea in pigs: An update on bacterial types, pathogenesis, and prevention strategies. Anim. Health Res. Rev..

[2] Hall A.L. (2017). Residue Monograph Prepared by the Meeting of the Joint FAO/WHO Expert Committee on Food Additives (JECFA). 85th Meeting 2017.

[3] Tummaruk P., Prapasarakul N. (2009). The use of herbal medicine as an alternative antimicrobial in the feed of post-weaning piglets: A field trial. J. Appl. Anim. Sci..

[4] Rezende L., Ferreira F., Pereira F., Figueiredo H., Guedes R. (2022). Evaluation of Nutritional Strategies in the Nursery Phase with Special Focus on the Use of Growth Promoters and Diet Complexity. SSRN Electron. J..

[5] Kandepu N., Kodaganur S.C., Mantri A.P., Saha S. (2012). RP-HPLC Method for Quantitative Estimation of Halquinol in Pharmaceutical Dosage Forms. Eurasian J. Anal. Chem..

[6] Van den Bogaard A.E., Hazen M., Hoyer M., Oostenbach P., Stobberingh E.E. (2002). Effects of flavophospholipol on resistance in fecal Escherichia coli and Enterococci of fattening pigs. Antimicrob. Agents Chemother..

[7] Lugsomya K., Tummaruk P., Hampson D.J., Prapasarakul N. (2012). Development of a modified selective medium to enhance the recovery rate of *Brachyspira hyodysenteriae* and other porcine intestinal spirochaetes from faeces. Lett. Appl. Microbiol..

[8] National Research Council (NRC) (2012). Nutrient Requirements of Swine.

[9] (2019). Official Methods of Analysis. http://www.eoma.aoac.org.

[10] (2017). *International Organization for Standardization* (ISO 6579-1:2017). Microbiology of the food chain-horizontal method for the detection, enumeration and serotyping of Salmonella-Part 1: Detection of *Salmonella* spp. Geneva, Switzerland. https://cdn.standards.iteh.ai/samples/56712/37da386eff674e07b35f9025371ee283/ISO-6579-1-2017.pdf.

[11] Pedersen K.N., Toft N. (2011). Intra-and inter-observer agreement when using a descriptive classification scale for clinical assessment of fecal consistency in growing pigs. Prev. Vet. Med..

[12] AOAC (2005). Official Methods of Analysis.

[13] Kongpanna P., Doerr J.A., Nilubol D., Jamikorn U. (2024). Effect of a multi-species direct-fed microbial on growth performance, nutrient digestibility, intestinal morphology and colonic volatile fatty acids in weanling pigs. Animals.

[14] Kozich J.J., Westcott S.L., Baxter N.T., Highlander S.K., Schloss P.D. (2013). Development of a dual-index sequencing strategy and curation pipeline for analyzing amplicon sequence data on the MiSeq Illumina sequencing platform. Appl. Environ. Microbiol..

[15] Caporaso J.G., Kuczynski J., Stombaugh J., Bittinger K., Bushman F.D., Costello E.K. (2010). QIIME allows analysis of high-throughput community sequencing data. Nat. Methods.

[16] Barbosa K.A., Genova J.L., Pazdziora M.L., Hennig J.F., Azevedo L.B.D., Veiga B.R.D.M., Carvalho P.L.D.O. (2023). The role of dietary monoglycerides and tributyrin in enhancing performance and intestinal health function in nursery piglets. Ital. J. Anim. Sci..

[17] Grecco H.A., Amorim A.B., Saleh M.A., Tse M.L., Telles F.G., Miassi G.M., Berto D.A. (2018). Evaluation of growth performance and gastro-intestinal parameters on the response of weaned piglets to dietary organic acids. An. Acad. Bras..

[18] Diana A., Boyle L.A., Leonard F.C. (2019). Removing prophylactic antibiotics from pig feed: How does it affect their performance and health?. BMC Vet. Res..

[19] Engelsmann M.N., Nielsen T.S., Hedemann M.S., Krogh U., Nørgaard J.V. (2023). Effect of postweaning feed intake on performance, intestinal morphology, and the probability of diarrhoea in piglets. Animal.

[20] Bosi P. (2011). Feed supplemented with 3 different antibiotics improved food intake and decreased the activation of the humoral immune response in healthy weaned pigs but had differing effects on intestinal microbiota. J. Anim. Sci..

[21] Guevarra R., Lee J., Lee S., Seok M., Kim D., Kang B. (2019). Piglet gut microbial shifts early in life: Causes and effects. J. Anim. Sci. Biotechnol..

[22] Habib M.A., Haque M.A., Islam M.S., Liton M.R. (2019). Effect of dietary Halquinol supplementation on the productive performances, carcass traits and blood profile of Sonali chicken. Asian J. Med. Biol. Res..

[23] Wealleans A.L., Bierinckx K., di Benedetto M. (2021). Fats and oils in pig nutrition: Factors affecting digestion and utilization. Anim. Feed Sci. Technol..

[24] Zou F., Qiu Y., Huang Y., Zou H., Cheng X., Niu Q. (2021). Effects of short-chain fatty acids in inhibiting HDAC and activating p38 MAPK are critical for promoting B10 cell generation and function. Cell Death Dis..

[25] Feehan B., Ran Q., Dorman V., Rumback K., Pogranichniy S., Ward K. (2022). Stability and volatility shape the gut bacteriome and *Kazachstania slooffiae* dynamics in preweaning, nursery and adult pigs. Sci. Rep..

[26] Neuman H., Forsythe P., Uzan A., Avni O., Koren O. (2018). Antibiotics in early life: Dysbiosis and the damage done. FEMS Microbiol. Rev..

[27] Abeles S.R., Jones M.B., Santiago-Rodriguez T.M., Ly M., Klitgord N., Yooseph S. (2016). Microbial diversity in individuals and their household contacts following typical antibiotic courses. Microbiome.

[28] Chen J., Mou L., Wang L., Wu G., Dai X., Chen Q., Tan Z. (2024). Mixed *Bacillus subtilis* and *Lactiplantibacillus plantarum*-fermented feed improves gut microbiota and immunity of Bamei pigletpigs. Front. Microbiol..

[29] Shabat S.K.B., Sasson G., Doron-Faigenboim A., Durman T., Yaacoby S., Miller M.E. (2016). Specific microbiome-dependent mechanisms underlie the energy harvest efficiency of ruminants. ISME J..

[30] Kim H.B., Isaacson R.E. (2015). The pig gut microbial diversity: Understanding the pig gut microbial ecology through the next generation high throughput sequencing. Vet. Microbiol..

[31] Huaman S.O.B., de Souza F.A., Bonato M.A., Dias C.P., Callegari M.A., Oba A., da Silva C.A. (2024). Effects of prebiotic and multispecies probiotic supplementation on the gut microbiota, immune function, and growth performance of weaned piglets. PLoS ONE.

[32] Turnbaugh P.J., Ley R.E., Mahowald M.A., Magrini V., Mardis E.R., Gordon J.I. (2006). An obesity-associated gut microbiome with increased capacity for energy harvest. Nature.

[33] Duarte M.E., Tyus J., Kim S.W. (2020). Synbiotic effects of enzyme and probiotics on intestinal health and growth of newly weaned pigs challenged with enterotoxigenic F18+ *Escherichia coli*. Front. Vet. Sci..

[34] Ding X., Lan W., Liu G., Ni H., Gu J.D. (2019). Exploring possible associations of the intestine bacterial microbiome with the pre-weaned weight gaining performance of piglets in intensive pig production. Sci. Rep..

[35] Kong Q., Zhang W., An M., Kulyar M.F.E.A., Shang Z., Tan Z., Liu S. (2022). Characterization of bacterial microbiota composition in healthy and diarrheal early-weaned Tibetan piglets. Front. Vet. Sci..

[36] Zhang W., Zhu Y.H., Zhou D., Wu Q., Song D., Dicksved J. (2017). Oral administration of a select mixture of bacillus probiotics affects the gut microbiota and goblet cell function following Escherichia coli challenge in newly weaned pigs of genotype MUC4 that are supposed to be enterotoxigenic *E. coli* F4ab/ac receptor. Appl. Environ. Microbiol..

[37] Irakli M., Lazaridou A., Biliaderis C.G. (2020). Comparative evaluation of the nutritional, antinutritional, functional, and bioactivity attributes of rice bran stabilized by different heat treatments. Foods.

[38] Su Y.T., Chen X.J., Liu M., Guo X.H. (2017). Effect of three lactobacilli with strain-specific activities on the growth performance, faecal microbiota and ileum mucosa proteomics of piglets. J. Anim. Sci. Biotechnol..

[39] Fouhse J.M., Zijlstra R.T., Willing B.P. (2016). The role of gut microbiota in the health and disease of pigs. Anim. Front..

[40] Wang X., Tsai T., Deng F., Wei X., Chai J., Knapp J. (2019). Longitudinal investigation of the swine gut microbiome from birth to market reveals stage and growth performance associated bacteria. Microbiome.

[41] Bin P., Tang Z., Liu S., Chen S., Xia Y., Liu J. (2018). Intestinal microbiota mediates enterotoxigenic Escherichia coli-induced diarrhea in piglets. BMC Vet. Res..

[42] Rinninella E., Raoul P., Cintoni M., Franceschi F., Miggiano G.A.D., Gasbarrini A. (2019). What is the healthy gut microbiota composition? a changing ecosystem across age, environment, diet, and diseases. Microorganisms.

[43] Zhang W., Ma C., Xie P., Zhu Q., Wang X., Yin Y. (2019). Gut microbiota of newborn piglets with intrauterine growth restriction have lower diversity and different taxonomic abundances. J. Appl. Microbiol..

[44] Strandwitz P., Kim K.H., Terekhova D., Liu J.K., Sharma A., Levering J. (2019). GABA-modulating bacteria of the human gut microbiota. Nat. Microbiol..

[45] Bell A., Juge N. (2021). Mucosal glycan degradation of the host by the gut microbiota. Glycobiology.

[46] Wexler H.M. (2007). Bacteroides: The good, the bad, and the nitty-gritty. Clin. Microbiol. Rev..

[47] Mills S., Stanton C., Lane J.A., Smith G.J., Ross R.P. (2019). Precision nutrition and the microbiome, part i: Current state of the science. Nutrients.

[48] Vandeputte D., Falony G., Vieira-Silva S., Tito R.Y., Joossens M., Raes J. (2016). Stool consistency is strongly associated with gut microbiota richness and composition, enterotypes and bacterial growth rates. Gut.

[49] Kwon H.J., Lim J.H., Kang D., Lim S., Park S.J., Kim J.H. (2019). Is stool frequency associated with the richness and community composition of gut microbiota?. Intest. Res..

[50] Li D.H., Li Z.P., Yan Z., Zhou G.Z., Ren R.R. (2021). Fecal fusobacterium nucleatum harbored virulence gene fadA are associated with ulcerative colitis and clinical outcomes. Microb. Pathog..

[51] Schokker D., Zhang J., Zhang L.L., Vastenhouw S.A., Heilig H.G., Smidt H. (2014). Early-life environmental variation affects intestinal microbiota and immune development in new-born piglets. PLoS ONE.

[52] Liao S.F., Nyachoti M. (2017). Using Probiotics to Improve Swine Gut Health and Nutrient Utilization. Anim. Nutr..

[53] Ren W., Yu B., Yu J., Zheng P., Huang Z., Luo J., Luo Y. (2022). Lower abundance of Bacteroides and metabolic dysfunction are highly associated with the post-weaning diarrhea in piglets. Sci. China Life Sci..

